# A new route for the synthesis of 1-deazaguanine and 1-deazahypoxanthine

**DOI:** 10.3762/bjoc.18.172

**Published:** 2022-11-29

**Authors:** Raphael Bereiter, Marco Oberlechner, Ronald Micura

**Affiliations:** 1 Institute of Organic Chemistry, Center for Molecular Biosciences, Innsbruck (CMBI), Innrain 80-82, 6020 Innsbruck, Austria

**Keywords:** deazapurine, heterocycles, imidazopyridines, nucleoside, nucleotides, pyrrolopyrimidines, RNA atomic mutagenesis

## Abstract

Imidazopyridines and pyrrolopyrimidines are an important class of compounds in medicinal chemistry. They can also be considered as deaza-modified purine nucleobases, and as such have attracted a lot of interest recently in the context of RNA atomic mutagenesis. In particular, for 1-deazaguanine (c^1^G base), a significant increase in demand is apparent. Synthetic access is challenging and the few reports found in the literature suffer from the requirement of hazardous intermediates and harsh reaction conditions. Here, we report a new six-step synthesis for c^1^G base, starting from 6-iodo-1-deazapurine. The key transformations are copper catalyzed C–O-bond formation followed by site-specific nitration. A further strength of our route is divergency, additionally enabling the synthesis of 1-deazahypoxanthine (c^1^I base).

## Introduction

Deazapurines (imidazopyridines and pyrrolopyrimidines) are N-heterocycles that have become an indispensable part of research in medicinal chemistry [1–3]. Especially, derivatives of 3-deazaguanine (imidazo[4,5-*c*]pyridines) [4], 7-deazaguanine/-hypoxanthine (pyrrolo[2,3-*d*]pyrimidines) [5–6], and 9-deazaguanine/-hypoxanthine (pyrrolo[3,2-*d*]-pyrimidines) [7–9] have been in the center of attention and were found to be effective compounds for the inhibition of various molecular targets associated with dysfunction of the central nervous system (e.g., as GABA and serotonin receptor modulators, or as inhibitors of phosphodiesterase PDE10A, glycogen synthase kinase 3 (GSK-3), leucine-rich repeat kinase 2 (LRRK2), tyrosine phosphorylation-regulated kinase-1A (DYRK1A) and CDC2-like kinase 1 (CLK1), and fatty acid amide hydrolase (FAAH) [4]). Similar properties were ascertained for 1-deazapurine derivatives (imidazo[4,5-*b*]pyridines) and nucleosides thereof [10–12], mostly associated with the inhibition of adenosine deaminase (ADA) [11] and as adenosine receptor antagonists [10]. Another important field of applications for deaza-modified nucleobases is their use in atom-specific mutagenesis experiments. For example, site specific 1-, 3-, and 7-deazapurine mutations of RNA have been fundamental to shed light on their structure, catalysis, and function [13–15]. However, difficulties in these fields arise from the lack of efficient synthetic protocols for various deaza-nucleosides and nucleobases. This is particularly true for the synthesis of 1-deazaguanine and 1-deazahypoxanthine. Previously published routes toward these compounds focused on ring closure of the imidazole part of the purine system after the pyrimidine core had been functionalized, however, the drawback of this approach is the passage of rather hazardous/explosive intermediates. Here, we present a new tactic for the syntheses of 1-deazaguanine and 1-deazahypoxanthine stimulated by a recently published route of our research group for the corresponding nucleosides [16–17], employing the same key reaction, namely the copper-catalyzed coupling of an aryl iodide with benzyl alcohol. We build on a commercially available imidazopyridine derivative and conceived a protecting group strategy to enhance solubility and selectivity to orchestrate the installation of the exocyclic amino and hydroxy groups.

## Results and Discussion

### 1-Deazaguanine

#### Previously described syntheses for 1-deazaguanine

**Scheme 1:**
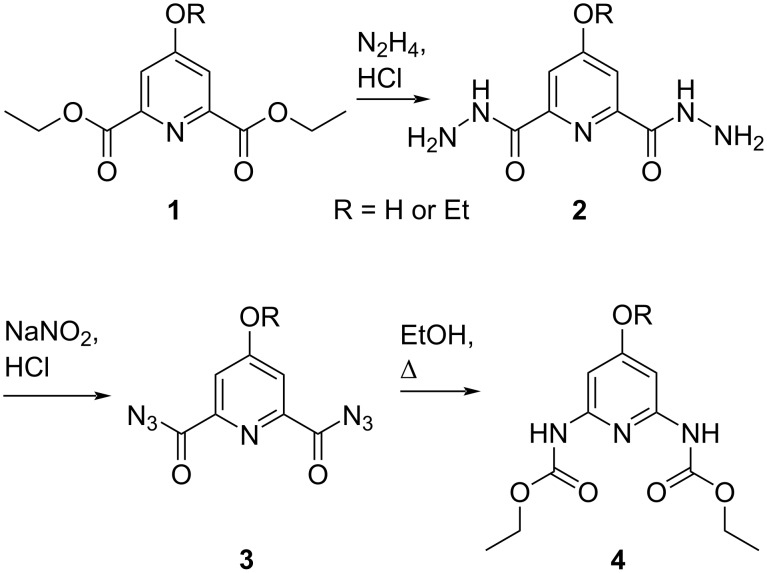
Syntheses of C4-substituted diethyl 2,6-pyridinedicarbamates **4**, passing hazardous and explosive diacylazide intermediates **3** that are required for Curtius rearrangement in the final step [19].

In 1956, Markees and Kidder reported the first access to 1-deazaguanine [18], followed by a comparable approach one year later by Gorton and Shive [19]. Both started with the conversion of diethyl chelidamate **1** to its dicarbamate analogue **4** (Scheme 1) and further accomplished their syntheses through different formations of the 1-deazapurine heterocycle. The syntheses of the diethyl 2,6-pyridinedicarbamate precursors via Curtius rearrangement, however, involved explosive chelidamyl diazide intermediates **3** (Scheme 1) [18–19].

**Scheme 2:**
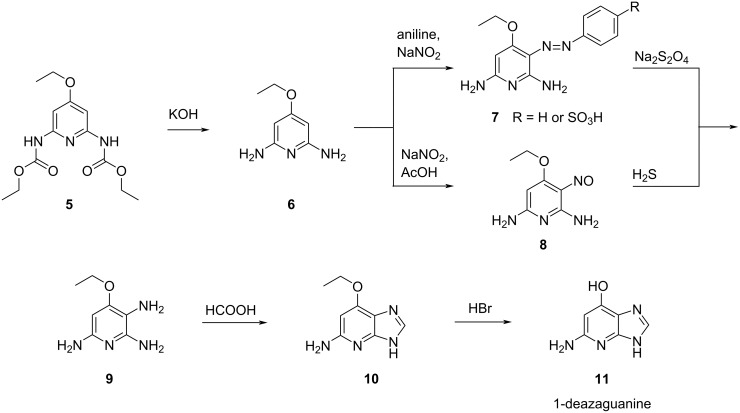
Synthesis of 1-deazaguanine (**11**) described by Markees and Kidder in 1956 [18].

Markees and Kidder used an ethyl protection for the O^6^ and described two options for the generation of 4-ethoxy-2,3,6-triaminopyridine (**9**). One possibility comprised the deprotection of the dicarbamate **5** with potassium hydroxide giving the diamine **6**, followed by azo coupling with the diazonium salt obtained from aniline and sodium nitrite to give azo compound **7**. Subsequent reduction with sodium dithionite then afforded triamine **9**. Another way comprised the installation of a nitroso group in compound **6** through reaction with in situ-generated nitrous acid giving nitroso compound **8**. The subsequent reduction to the corresponding amine with hydrogen sulfide afforded the desired triamine **9**. After cyclization of the resulting 4-ethoxy-2,3,6-triaminopyridine (**9**) with formic acid leading to ethyl-protected compound **10** and liberation of the O^6^ with hydrogen bromide, 1-deazaguanine (**11**) was formed in 2 to 4% overall yield (Scheme 2) [18].

**Scheme 3:**
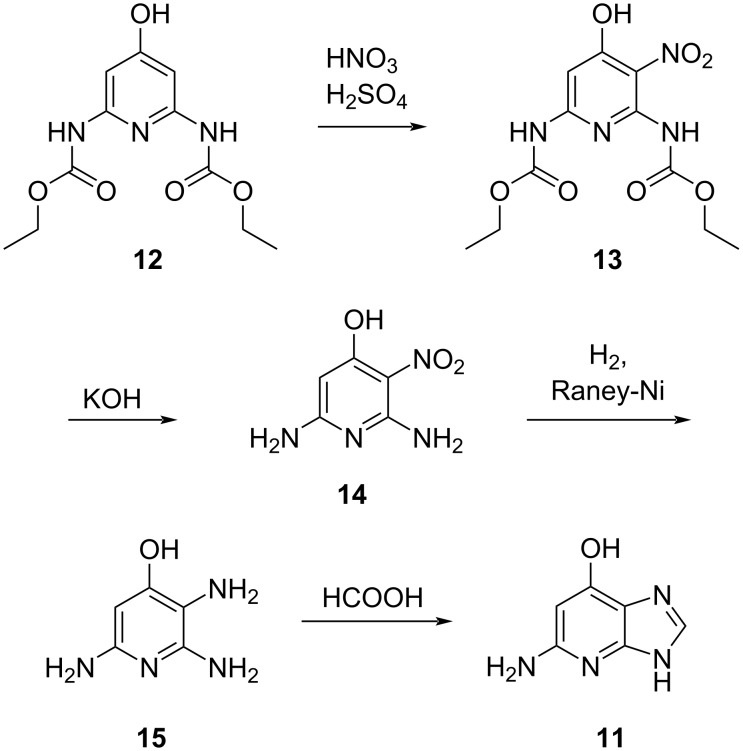
Synthesis of 1-deazaguanine (**11**) described by Gorton and Shive in 1957 [19].

The approach by Gorton and Shive differed from the above path by leaving the hydroxy group of all intermediate 4-hydroxypyridine derivatives **12**–**15** unprotected [19]. Moreover, instead of azo coupling or nitroso formation, a simple nitration protocol with nitric acid to give the nitro derivative **13** and subsequent reduction with Raney nickel was carried out to obtain the desired 4-hydroxy-2,3,6-triaminopyridine (**15**) in unspecified yield (Scheme 3). This approach was optimized in 1975 by Schelling and Salemink using benzyl ether protection of the O^4^ during the imidazopyridine formation to increase the overall yield up to 37% [20]. The last attempt to refine the synthesis of Gorton and Shive, was described by Temple and co-workers in 1976 by their preparation of 1-deaza-6-thioguanine analogues with 28% overall yield [21].

#### Synthesis of 1-deazaguanine

**Scheme 4:**
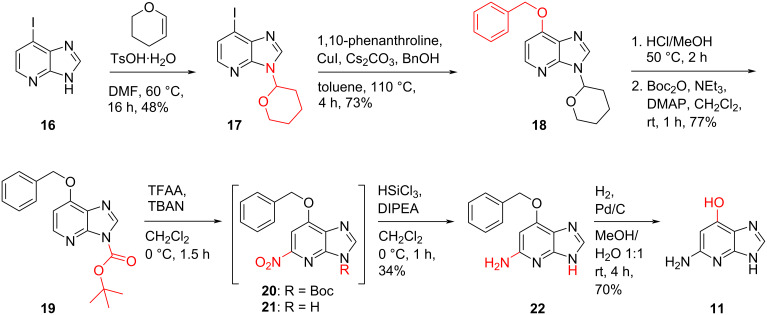
Six-step synthesis of 1-deazaguanine (**11**). Abbreviations: *p*-toluenesulfonic acid (TsOH), 4-(dimethylamino)pyridine (DMAP), trifluoroacetic anhydride (TFAA), tetrabutylammonium nitrate (TBAN), *N*,*N*-diisopropylethylamine (DIPEA).

Our route to 1-deazaguanine **11** started from 6-iodo-1-deazapurine (**16**) (Scheme 4), which can be easily prepared from its commercially available 6-chloro derivative [16]. To enable C–O coupling with benzyl alcohol, protection of the N9 with a tetrahydropyranyl group was necessary due to limited solubility of the aryl iodide. Therefore, 6-iodo-1-deazapurine was treated with tosylic acid and 3,4-dihydropyran in dimethylformamide to obtain the corresponding tetrahydropyranyl-protected amine **17**. Subsequently, a copper-catalyzed C–O bond formation at C6 using benzyl alcohol in the presence of caesium carbonate, copper(I) iodide, and 1,10-phenanthroline furnished benzyl ether **18** in excellent yields [22]. The switch from 2-tetrahydropyranyl to *tert*-butyloxycarbonyl protected compound **19** was required for an efficient installation of the exocyclic amine via regioselective nitration in the presence of trifluoroacetic anhydride (TFAA) and tetrabutylammonium nitrate (TBAN) to give a mixture of the *tert*-butyloxycarbonyl-protected and deprotected 2-nitro-intermediates **20** and **21**, respectively [23–25]. The nitro group was then selectively reduced using trichlorosilane and *N*,*N*-diisopropylethylamine (DIPEA) to form benzyl-protected 1-deazaguanine **22** [26]. Finally, the *O*^6^-benzyl moiety was cleaved under Pd/C-catalyzed hydrogenation to provide 1-deazaguanine (**11**) in six steps and 6% overall yield. A total of 120 mg of **11** were obtained in the course of this study. At this point, we note that *N*9-*tert*-butyloxycarbonyl-protected 6-iodo-1-deazapurine was successfully synthesized but not stable during the cross coupling reaction. We also mention that we did not decide for a direct transformation [27–28] of 6-iodo-1-deazapurine into 6-hydroxy-1-deazapurine for reasons of solubility and desired regioselectivity of the subsequent nitration reaction.

### 1-Deazahypoxanthine

#### Synthesis of 1-deazahypoxanthine

**Scheme 5:**
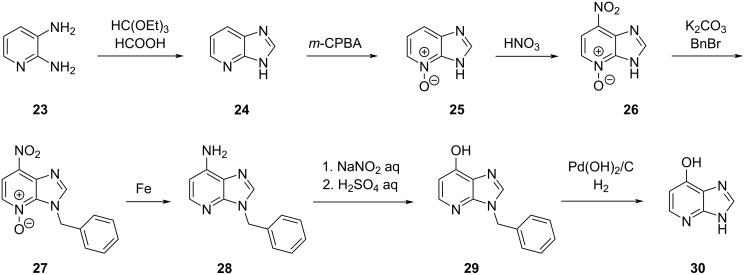
1-Deazahypoxanthine (**30**) synthesis described by Kubo and Hirao in 2019 [29]. For reason of simplicity only one regioisomer of the N7/N9 benzyl compound mixtures is shown.

To the best of our knowledge, only one synthesis of 1-deazahypoxanthine has been described so far [29]. Kubo and Hirao started their synthesis from 2,3-diaminopyridine (**23**) which was converted into benzyl-protected 1-deazaadenine **28** (as a mixture of N7 and N9 regioisomers) in five steps, followed by the introduction of a hydroxy group at C6 under Sandmeyer conditions to give **29** (Scheme 5). To remove the benzyl group, Pearlman’s catalyst in the presence of hydrogen was applied to provide 1-deazahypoxanthine (**30**) in seven steps and 24% overall yield.

**Scheme 6:**
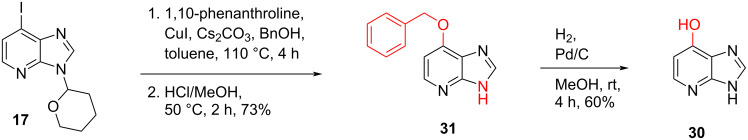
Synthesis of 1-deazahypoxanthine (**30**).

Our new route to 1-deazahypoxanthine (**30**) starts from the tetrahydropyranyl-protected 6-iodo-1-deazapurine **17** which was converted into the *O*^6^-benzyl derivative **31** using the copper-catalyzed C–O-bond formation as described above (Scheme 6). Without purification the crude product was treated with hydrochloric acid in methanol to remove the tetrahydropyranyl protecting group. The final step was then accomplished by hydrogenation of benzyl ether **31** to obtain 1-deazahypoxanthine (**30**) in 44% overall yield.

## Conclusion

We have developed convenient synthetic routes for 1-deazaguanine (**11**) and 1-deazahypoxanthine (**30**). Starting from readily accessible 6-iodo-1-deazapurine [16], the key reactions are copper-catalyzed benzyl ether formation and site-specific nitration. The application of protecting groups was necessary for reasons of solubility and to improve selectivity. The obtained heterocycles may serve as core compound for further structural diversification and applications in medicinal chemistry. They will also be useful for nucleosidation reactions to prepare the corresponding nucleosides in straightforward manner.

## Experimental

**General.** Chemical reagents and solvents were purchased in the highest available quality from commercial suppliers (Merck/Sigma-Aldrich, ABCR, Synthonix) and used without further purification. Analytical thin-layer chromatography (TLC) was performed on Macherey-Nagel Polygram^®^ SIL G/UV254 plates. 0.2 mm Silica gel 60 for column chromatography was purchased from Macherey-Nagel. ^1^H and ^13^C NMR spectra were recorded on a Bruker UltrashieldTM 400 MHz Plus or a 700 MHz Avance Neo spectrometer. Chemical shifts (δ) are reported relative to tetramethylsilane (TMS), referenced to the residual solvent signal (DMSO-*d*_6_: 2.50 ppm for ^1^H and 39.52 ppm for ^13^C spectra; CDCl_3_: 7.26 ppm for ^1^H and 77.16 ppm for ^13^C spectra). Signal assignments are based on ^1^H,^1^H-COSY, ^1^H,^13^C-HSQC and ^1^H,^13^C-HMBC experiments. High resolution mass spectra were recorded in positive ion mode unless otherwise noted on a Thermo Scientific Q Exactive Orbitrap.

### 6-Iodo-9-(tetrahydro-2*H*-pyran-2-yl)-1-deazapurine (**17**)

6-Iodo-1-deazapurine (2.89 g, 11.79 mmol), *p*-toluenesulfonic acid monohydrate (TsOH·H_2_O, 0.34 g, 1.77 mmol) and 3,4-dihydro-2*H*-pyran (2.98 g, 3.21 mL, 35.38 mmol) were dissolved in 5 mL dry *N*,*N*-dimethylformamide and stirred overnight for 16 hours at 60 °C. The reaction was quenched by adding ammonia 25% (4 mL) and was stirred for further 5 minutes. The solvent and all volatiles were removed under reduced pressure and the oily residue was dissolved in dichloromethane containing 4% triethylamine. Subsequently, the mixture was washed three times with brine, dried over Na_2_SO_4_, filtered, and concentrated to dryness. The crude product was purified via silica gel chromatography using 20 to 50% ethyl acetate in cyclohexane (containing 2% triethylamine) as gradient. Yield: 1.86 g (48%) of compound **17** as a brownish solid. TLC (ethyl acetate/cyclohexane 1:1, 2% NEt_3_): *R*_f_ 0.38; ^1^H NMR: (400 MHz, DMSO-*d*_6_, 25 °C) δ 1.58 (m, 2H, *H*2-C(5)-pyran), 1.75 (m, 1H, *H*(b)-C(4)-pyran), 1.98 (m, 2H, *H*(b)-C(3)-pyran & *H*(a)-C(4)-pyran), 2.31 (m, 1H, *H*(a)-C(3)-pyran), 3.70 (m, 1H, *H*(b)-C(6)-pyran), 4.01 (m, 1H, *H*(a)-C(6)-pyran), 5.76 (q, *J* = 4.29 Hz, 1H, *H*-C(2)-pyran), 7.78 (d, *J* = 5.04 Hz, 1H, *H*-C(1)), 8.05 (d, *J* = 5.04 Hz, 1H, *H*-C(2)), 8.75 (s, 1H, *H*-C(8)); ^13^C NMR: (400 MHz, DMSO-*d*_6_, 25 °C) δ 22.46 (H-*C*(3)-pyran), 24.52 (H-*C*(4)-pyran), 29.94 (H-*C*(5)-pyran), 67.73 (H-*C*(6)-pyran), 81.23 (H-*C*(2)-pyran), 99.35 (H-*C*(6)), 127.76 (H-*C*(1)), 137.84 (H-*C*(8)), 143.30 (H-*C*(5)), 144.07 (H-*C*(4)), 144.23 (H-*C*(2)); ESIMS (*m*/*z*): [M + H]^+^ calcd for 330.01; found, 330.01.

### 6-Benzyloxy-9-(tetrahydro-2*H*-pyran-2-yl)-1-deazapurine (**18**)

Compound **17** (1.72 g, 5.23 mmol), copper(I) iodide (CuI, 99.52 mg, 0.52 mmol), 1,10-phenanthroline (188.35 mg, 1.05 mmol), caesium carbonate (Cs_2_CO_3_, 2.38 g, 7.32 mmol) and benzyl alcohol (BnOH, 1.13 g, 1.07 mL, 10.45 mmol) were suspended in 2.62 mL toluene (0.5 mL per 1 mmol compound **17**) and stirred for 4 hours at 110 °C under atmospheric conditions. After complete reaction (TLC reaction control), the suspension was allowed to cool to room temperature and the catalyst was filtered through celite and washed with dichloromethane containing 1% triethylamine. The filtrate was concentrated to dryness and the crude product was purified via silica gel chromatography using 20 to 40% ethyl acetate in cyclohexane (containing 1% triethylamine) as gradient. Yield: 1.18 g (73%) of compound **18** as a brownish solid. TLC (cyclohexane/ethyl acetate 1:1): *R*_f_ 0.23; ^1^H NMR (400 MHz, DMSO-*d*_6_, 25 °C) δ 1.57 (m, 2H, *H_2_*-C(5)-pyran), 1.74 (m, 1H, *H(b)*-C(4)-pyran), 1.95 (m, 2H, *H(b)*-C(3)-pyran & *H(a)*-C(4)-pyran), 2.29 (m, 1H, *H(a)*-C(3)-pyran), 3.69 (m, 1H, *H(b)*-C(6)-pyran), 4.01 (m, 1H, *H(a)*-C(6)-pyran), 5.53 (s, 2H, *H_2_*C-(benzyl)), 5.76 (d, *J* = 4.38 Hz, 1H, *H*-C(2)-pyran), 6.96 (d, *J* = 5.04 Hz, 1H, *H*-C(1)), 7.34–7.51 (5H, *H*C-arom. (benzyl)), 8.21 (d, *J* = 5.04 Hz, 1H, *H*-C(2)), 8.50 (s, 1H, *H*-C(8)); ^13^C NMR (100 MHz, DMSO-*d*_6_, 25 °C) δ 22.58 (H-*C*(3)-pyran), 24.57 (H-*C*(4)-pyran), 30.10 (H-*C*(5)-pyran), 67.69 (H-*C*(6)-pyran), 70.51 (H_2_*C*-benzyl), 80.77 (H-*C*(2)-pyran), 103.74 (H-*C*(1)), 124.73 (H-*C*(5)), 127.96, 128.14, 128.49 (H*C*-benzyl), 136.39 (*C*-quart.-benzyl), 140.79 (H-*C*(8)), 145.59 (H-*C*(2)), 148.17 (H-*C*(4)), 156.27 (H-*C*(6)); ESIMS (*m*/*z*): [M + H]^+^ calcd for 310.16; found, 310.16.

### 6-Benzyloxy-9-(*tert*-butyloxycarbonyl)-1-deazapurine (**19**)

Compound **18** (0.9 g, 2.91 mmol) was dissolved in methanol (20 mL), then 5% hydrochloric acid (5 mL) was added and stirred for 2 hours at 50 °C. After complete deprotection (TLC reaction control!), the solvent and all volatiles were removed under reduced pressure and the crude residue was suspended in dichloromethane (6.5 mL). Afterwards, di-*tert*-butyl dicarbonate (Boc_2_O, 888.89 mg, 4.07 mmol), 4-(dimethylamino)pyridine (DMAP, 35 mg, 0.29 mmol) and triethylamine (NEt_3_, 589 mg, 811 µL, 5.82 mmol) were added and the clear solution was stirred for one hour at room temperature. The mixture was subsequently quenched with saturated ammonium chloride solution, filtrated and concentrated to dryness. The crude product was purified via silica gel chromatography using 0 to 3 % methanol in dichloromethane as gradient. Yield: 730 mg (77%) of compound **19** as a brownish solid. TLC: (6% methanol in dichloromethane): *R*_f_ 0.70; ^1^H NMR (400 MHz, CDCl_3_, 25 °C) δ 1.69 (s, 9H, C(C*H_3_*)_3_-Boc), 5.51 (s, 2H, *H_2_*C-(benzyl)), 6.82 (d, *J* = 5.70 Hz, 1H, *H*-C(1)), 7.31–7.49 (5H, *H*C-arom. (benzyl)), 8.35 (s, 1H, *H*-C(8)), 8.36 (d, *J* = 5.70 Hz*,* 1H, *H*-C(2)); ^13^C NMR (100 MHz, CDCl_3_, 25 °C) δ 28.14 (C(*C*H_3_)_3_-Boc), 71.60 (H_2_*C*-benzyl), 86.18 (*C*(CH_3_)_3_-Boc), 105.44 (H-*C*(1)), 126.46 (H-*C*(5)), 127.71, 128.48, 128.81 (H*C*-benzyl), 135.89 (*C*-quart.-benzyl), 140.80 (H-*C*(8)), 146.50 (C=O, Boc), 148.08 (H-*C*(2) & H-*C*(4)), 157.22 (H-*C*(6)). ESIMS (*m*/*z*) [M + H]^+^ calcd for 326.15; found, 326.15.

### *O*^6^-Benzyl-1-deazaguanine (**22**)

Nitration: Tetrabutylammonium nitrate (TBAN, 624.64 mg, 2.05 mmol) was dissolved in 6 mL dry dichloromethane under argon atmosphere, then, trifluoroacetic anhydride (TFAA, 430.88 mg, 285.35 µL, 2.05 mmol) was added and the mixture was stirred at 0 °C for 10 minutes. Meanwhile, compound **19** (445.00 mg, 1.37 mmol) was dissolved in dry dichloromethane (6 mL) and cooled to 0 °C under argon atmosphere. The nitration mixture was transferred with a syringe and added over a period of 10 minutes to the dissolved substrate. The resulting reaction mixture was stirred for 1.5 hours at 0 °C under an argon atmosphere. Afterwards, the reaction was quenched by adding the whole reaction mixture into a separatory funnel containing saturated sodium bicarbonate solution, followed by vigorously shaking (do not use a stopper!). The organic layer was washed with saturated sodium bicarbonate solution and brine and was finally dried over Na_2_SO_4_, filtered and concentrated to dryness. The crude product was purified via silica gel chromatography using 0 to 4% methanol in dichloromethane as gradient. Yield: 395 mg of a mixture containing the 2-nitro-compound **20** and **21** (for NMR spectra see Supporting Information File 1). TLC: (5% acetone in toluene): *R*_f_ 0.34 (compound **20**), *R*_f_ 0.05 (compound **21**).

Reduction: The mixture from the above procedure was suspended in dry dichloromethane (8 mL) at 0 °C, and *N*,*N*-diisopropylethylamine (DIPEA, 1.06 g, 1.43 mL, 8.21 mmol) was added. A solution containing dry dichloromethane (4 mL) and trichlorosilane (HSiCl_3_, 778 mg, 581 µL, 5.74 mmol) was prepared at 0 °C under an argon atmosphere, and then added via syringe to the substrate. The mixture was stirred at 0 °C for one hour. Subsequent quenching was achieved by the addition of saturated bicarbonate solution (10 mL) and further stirring for one hour. Afterwards, two spoons of silica were added and the whole suspension was concentrated to dryness and dried under high vacuum until a fine powder remains. The silica adsorbed with the product was loaded onto a short silica column and the product was eluted using 0 to 20% methanol in dichloromethane as gradient. The brownish solid was dissolved in boiling water, filtered and cooled to room temperature to precipitate a white solid. Yield: 110 mg (34%, over two steps) of compound **22** as a white solid. TLC: (15% methanol in dichloromethane): *R*_f_ 0.44; ^1^H NMR (400 MHz, DMSO-*d*_6_, 25 °C) δ 5.34 (s, 2H, *H_2_*C-(benzyl)), 6.10 (s, 1H, *H*-C(1)), 7.36–7.52 (5H, *H*C-arom. (benzyl)), 7.93 (s, 1H, *H*-C(8)); ^13^C NMR (100 MHz, DMSO-*d*_6_, 25 °C) δ 71.03 (H_2_*C*-benzyl), 88.06 (H-*C*(1)), 110.84 (H-*C*(5)), 128.30, 128.38, 128.71 (H*C*-benzyl), 135.11 (*C*-quart.-benzyl), 140.84 (H-*C*(4)), 145.69 (H-*C*(8)), 155.63 (H-*C*(2)), 157.47 (H-*C*(6)); ESIMS (*m*/*z*): [M + H]^+^ calcd for 241.11; found, 241.11.

### 1-Deazaguanine (**11**)

Compound **22** (111 mg, 462 µmol) and palladium on carbon 10% (Pd/C, 180.56 mg) were suspended in H_2_O/methanol 1:1 (7 mL). A rubber septum was applied and hydrogen gas (balloon with syringe) was bubbled through the solution for 10 minutes. The mixture was stirred under hydrogen atmosphere for further four hours at room temperature. The catalyst was filtered through celite and the filtrate was concentrated to dryness. The crude product was purified via silica gel chromatography using 0 to 25% methanol in dichloromethane as gradient. Yield: After recrystallization from water 48 mg (70%) of compound **11** as a white solid. TLC: (25% methanol in dichloromethane) *R*_f_ 0.19; ^1^H NMR (400 MHz, DMSO-*d*_6_, 25 °C) δ 5.43 (s, 1H, *H*-C(1)), 5.76 (b, 2H, N*H_2_*), 7.78 (s, 1H, *H*-C(8)), 11.69 (b, 2H, N*H* & O*H*); ^13^C NMR (100 MHz, DMSO-*d*_6_, 25 °C) δ 89.87 (H-*C*(1)), 113.28 (H-*C*(5)), 139.82 (H-*C*(8)), 145.69 (H-*C*(4)), 154.08 (H-*C*(2)), 162.52 (H-*C*(6)); ESIMS (*m*/*z*): [M + H]^+^ calcd for 151.06; found, 151.06.

### *O*^6^-Benzyl-1-deazahypoxanthine (**31**)

Compound **17** (500 mg, 1.52 mmol), copper(I) iodide (CuI, 28.93 mg, 152 µmol), 1,10-phenanthroline (54.75 mg, 304 µmol), caesium carbonate (Cs_2_CO_3_, 692.93 mg, 2.13 mmol) and benzyl alcohol (BnOH, 328.55 mg, 310 µL, 3.04 mmol) were suspended in 0.76 mL of toluene (0.5 mL per 1 mmol compound **17**) and stirred for 4 hours at 110 °C under atmospheric conditions. After complete reaction (TLC reaction control), the suspension was allowed to cool to room temperature and the catalyst was filtered through celite and washed with dichloromethane containing 1% triethylamine. The filtrate was concentrated to dryness and the remaining solid was dissolved in methanol (20 mL) and 5% hydrochloric acid (5 mL). This solution was stirred for two hours at 50 °C and concentrated to dryness. The crude compound was purified via silica gel chromatography using 0 to 10% methanol in dichloromethane as gradient. Yield: 250 mg (73%) of compound **31** as a white solid. TLC: (10% methanol in dichloromethane): *R*_f_ 0.44; ^1^H NMR (400 MHz, DMSO-*d*_6_, 25 °C) δ 5.62 (s, 2H, *H_2_*C-(benzyl)), 5.76 (d, *J* = 4.38 Hz, 1H, *H*-C(2)-pyran), 7.39–7.62 (6H, *H*C-arom. (benzyl) & *H*-C(1)), 8.63 (d, *J* = 6.60 Hz, 1H, *H*-C(2)), 8.91 (s, 1H, *H*-C(8)); ^13^C NMR (400 MHz, DMSO-*d*_6_, 25 °C) δ 71.89 (H_2_*C*-benzyl), 103.56 (H-*C*(1)), 118.33 (H-*C*(5)), 128.29, 128.66 (H*C*-benzyl), 134.85 (*C*-quart.-benzyl), 140.65 (H-*C*(2)), 145.81 (H-*C*(8)), 148.62 (H-*C*(4)), 157.10 (H-*C*(6)); ESIMS (*m*/*z*): [M + H]^+^ calcd for 226.10; found, 226.10.

### 1-Deazahypoxanthine (**30**)

Compound **31** (230 mg, 1.02 mmol) was dissolved in methanol (15 mL), then palladium on charcoal 10% (Pd/C, 400 mg, 337 µmol) was added and hydrogen (balloon with syringe and septum) was bubbled through the suspension for 10 minutes. Subsequently, the mixture was vigorously stirred under hydrogen atmosphere for 4 hours at room temperature. Afterwards, the suspension was filtered through celite, and the filtrate was concentrated to dryness. The remaining crude product was purified by recrystallization in water. Yield: 83 mg (60%) of compound **30** as a white solid. ^1^H NMR (700 MHz, DMSO-*d*_6_* +* 40 µL 5% HCl, 25 °C) δ 6.96 (d, *J* = 6.83 Hz, 1H, *H*-C(1)), 8.26 (d, *J* = 6.83 Hz, 1H, *H*-C(2)), 8.58 (s, 1H, *H*-C(8)); ^13^C NMR (175 MHz, DMSO-*d*_6_* +* 40 µL 5% HCl, 25 °C) δ 107.00 (H-*C*(1)), 118.88 (H-*C*(5)), 137.87 (H-*C*(2)), 145.78 (H-*C*(8)), 149.08 (H-*C*(4)), 160.86 (H-*C*(6)); ESIMS (*m*/*z*): [M − H]^−^ calcd for 134.04; found, 134.03.

## Supporting Information

File 1 NMR spectra of compounds **17** to **22**, **11**, **31** and **30**.
