# Supplementary material for: A new route for the synthesis of 1-deazaguanine and 1-deazahypoxanthine
Source: Beilstein J Org Chem. 2022 Nov 29;18:1617–24. doi: 10.3762/bjoc.18.172 (PMC9727274; doi:10.3762/bjoc.18.172)

BEILSTEIN JOURNAL OF ORGANIC CHEMISTRY

Supporting Information

for

A new route for the synthesis of 1-deazaguanine and 1-deazahypoxanthine

Raphael Bereiter, Marco Oberlechner and Ronald Micura

Beilstein J. Org. Chem. **2022,** *18,* 1617–1624. doi:10.3762/bjoc.18.172

NMR spectra of compounds 17 to 22, 11, 31 and 30

License and Terms: This is a supporting information file under the terms of the Creative Commons Attribution License (https://creativecommons.org/licenses/by/4.0). Please note that the reuse, redistribution and reproduction in particular requires that the author(s) and source are credited and that individual graphics may be subject to special legal provisions.
The license is subject to the *Beilstein Journal of Organic Chemistry* terms and conditions: (https://www.beilstein-journals.org/bjoc/terms)

Table of contents

NMR spectra of compounds **17** to **22**, **11**, **31** and **30** S2–S12

All synthetic procedures are described in the main text.

NMR spectra of compounds 17 to 22, 11, 31 and 30

^{1}H NMR (400 MHz, DMSO-d_6, 25 °C) of compound **17**

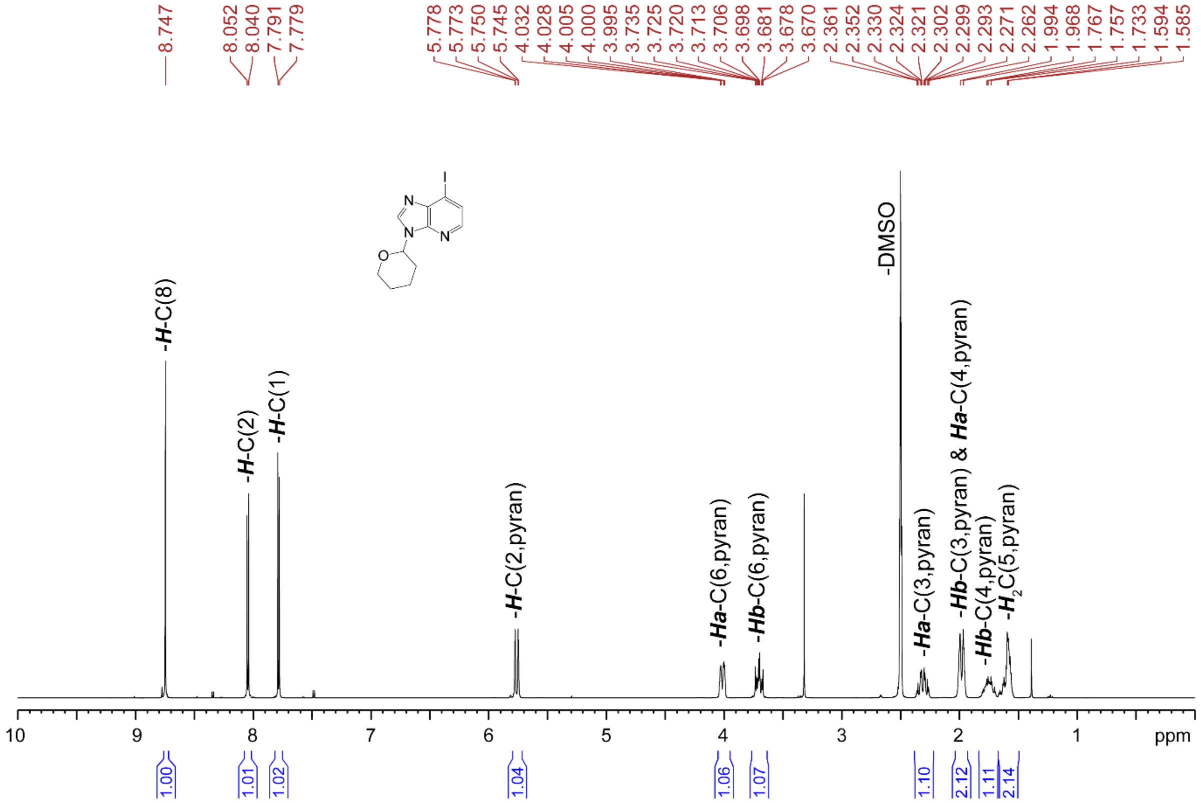

^{13}C NMR (100 MHz, DMSO-d_6, 25 °C) of compound **17**

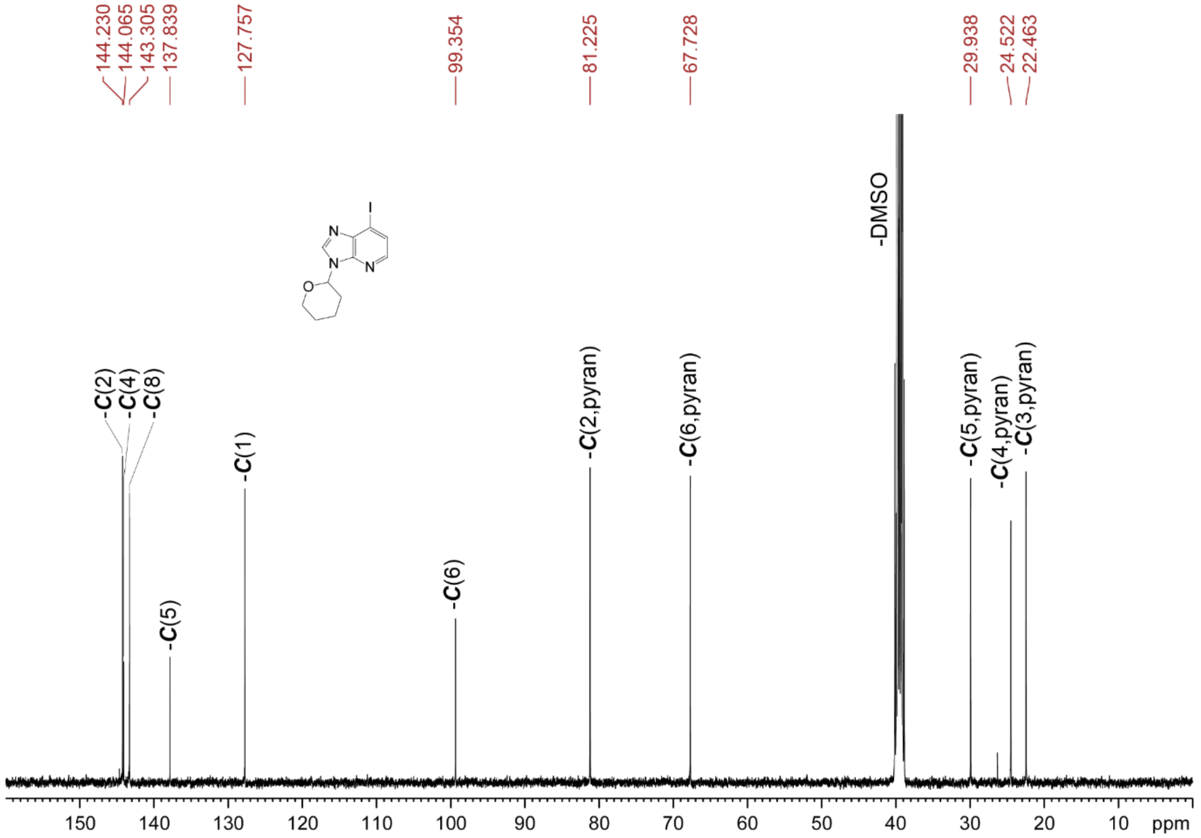

^{1}H NMR (400 MHz, DMSO-d_6, 25 °C) of compound **18**

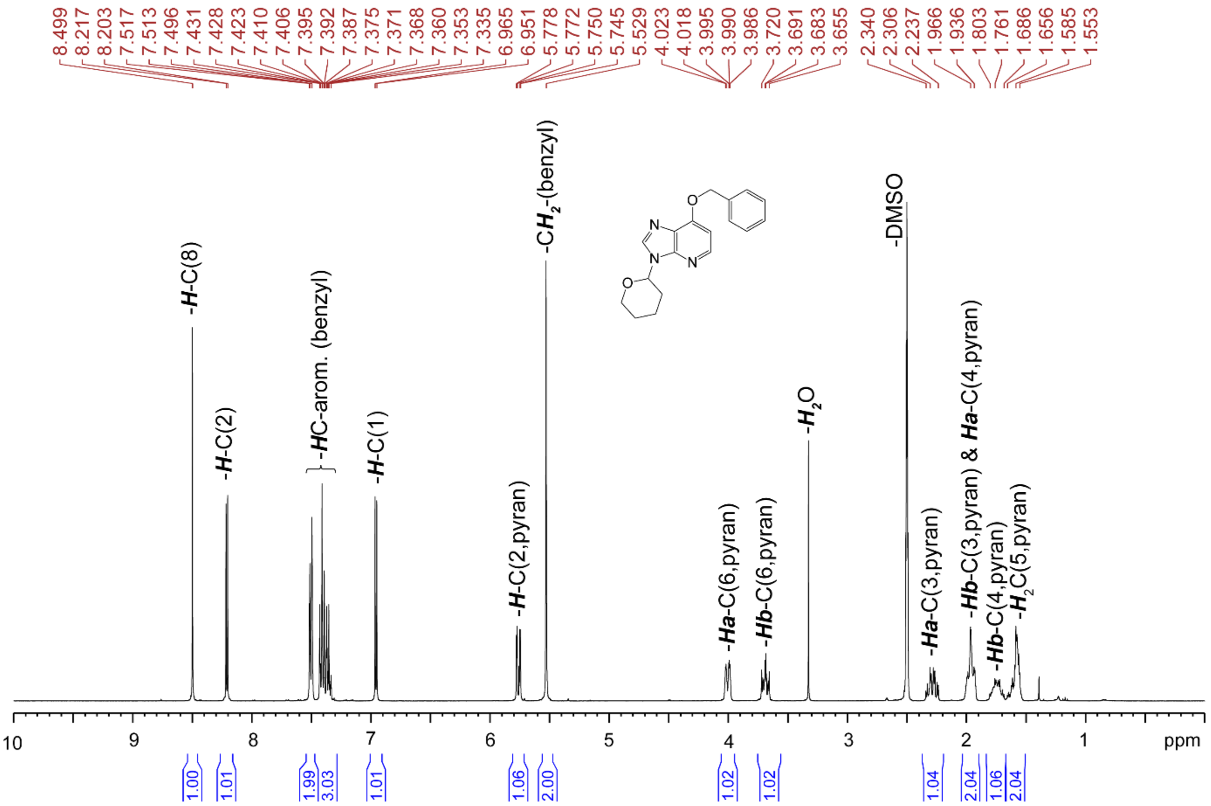

^{13}C NMR (100 MHz, DMSO-d_6, 25 °C) of compound **18**

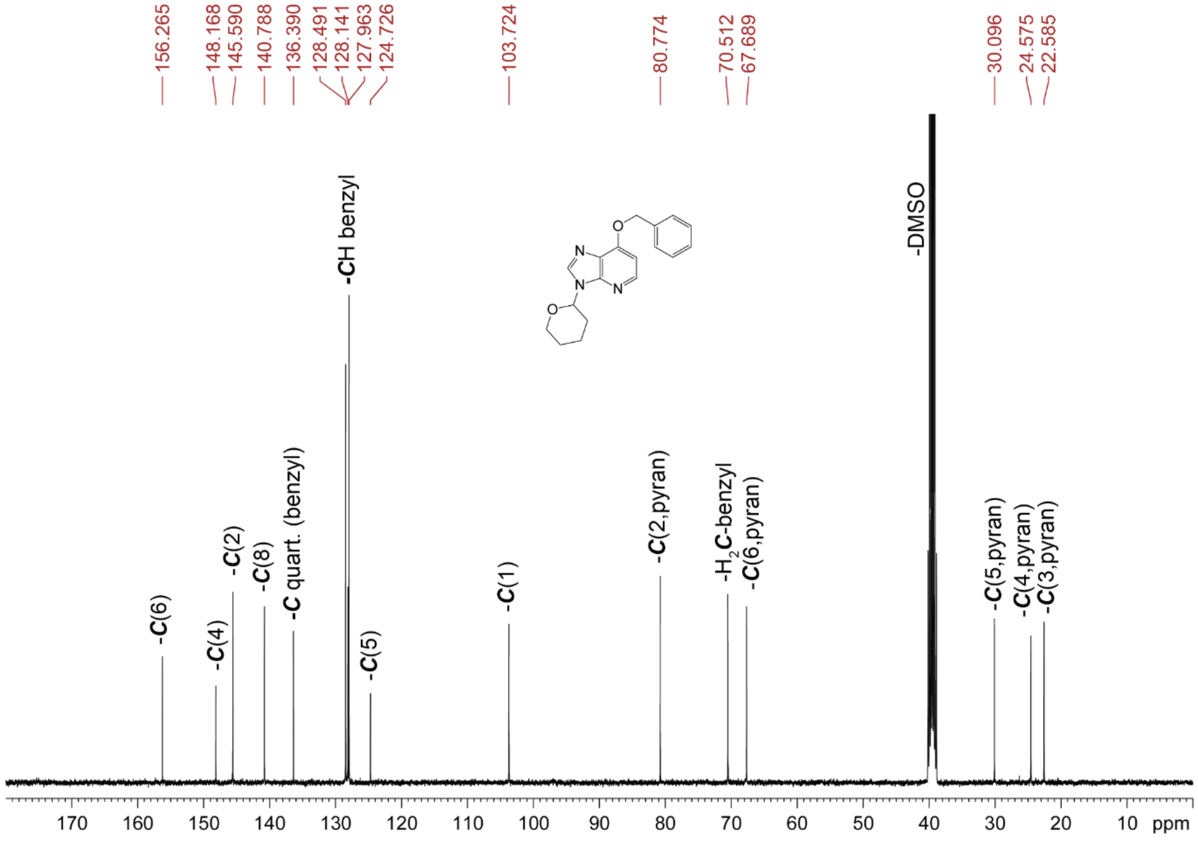

^{1}H NMR (400 MHz, $CDCl_3$, 25 °C) of compound **19**

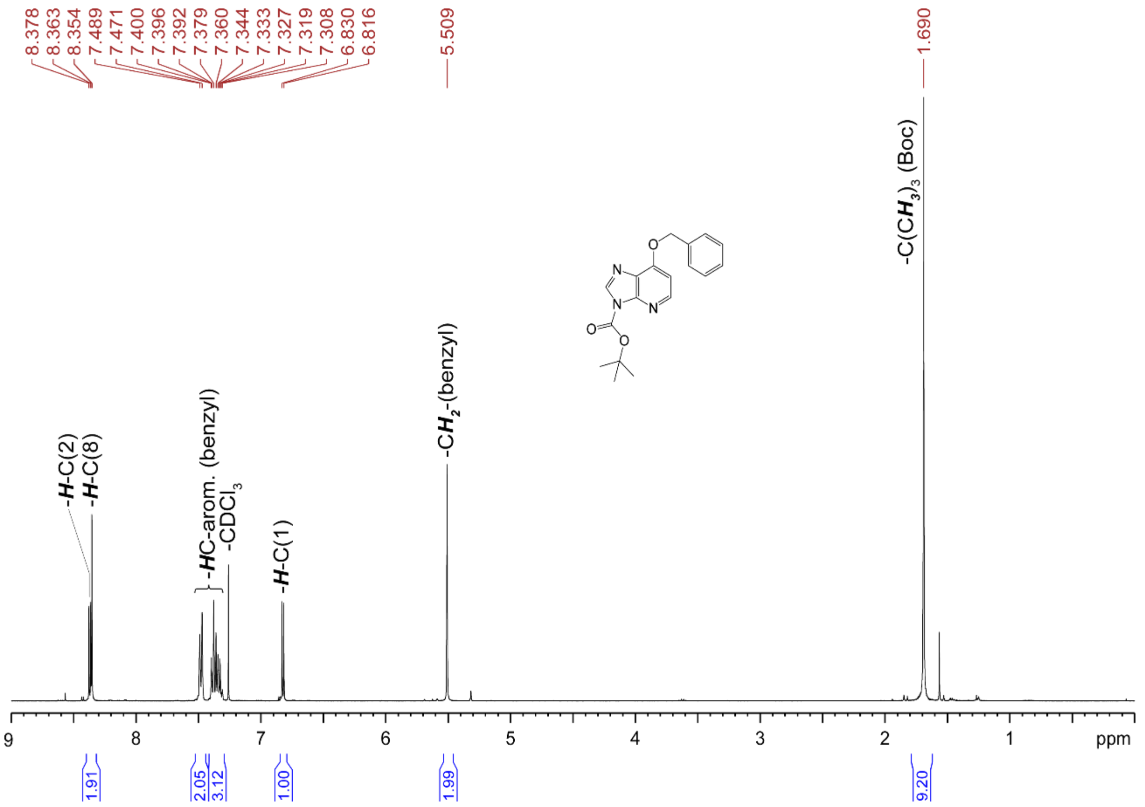

^{13}C NMR (100 MHz, $CDCl_3$, 25 °C) of compound **19**

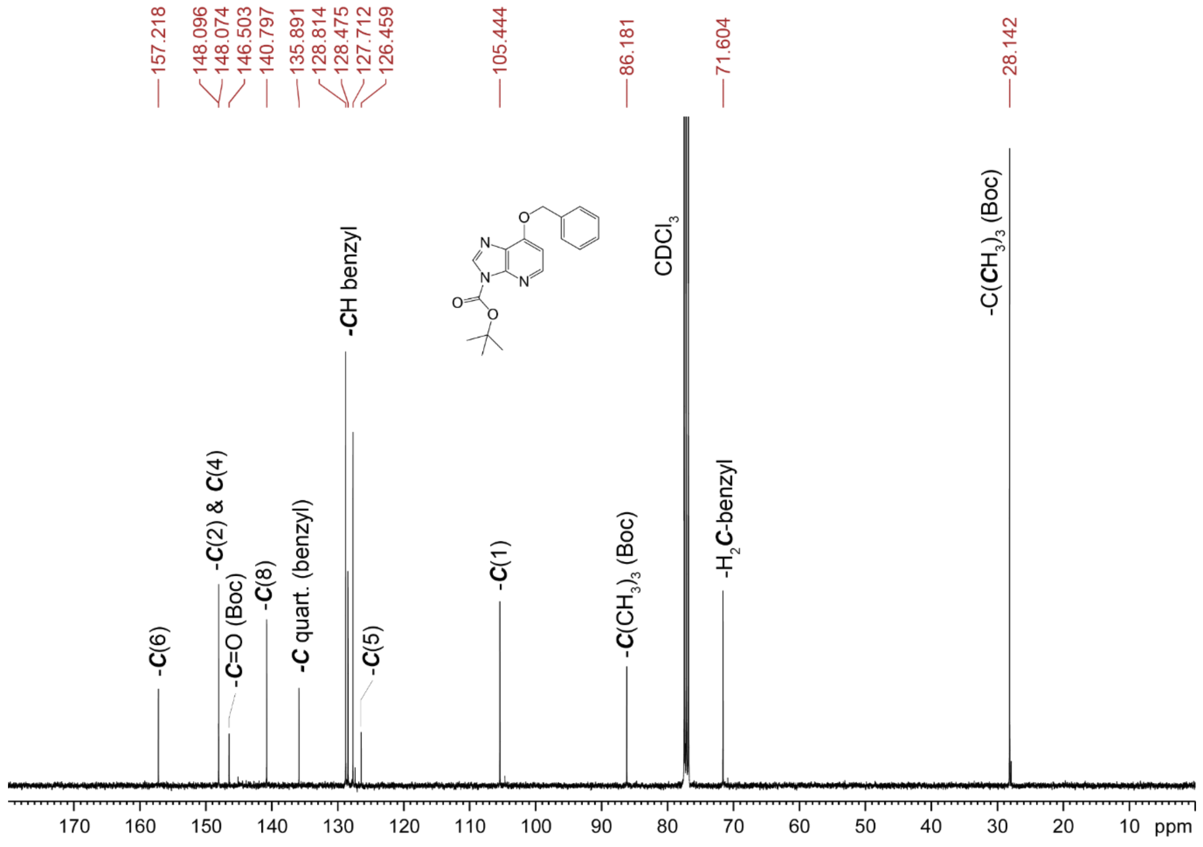

^{1}H NMR (400 MHz, $CDCl_3$, 25 °C) of compound **20**

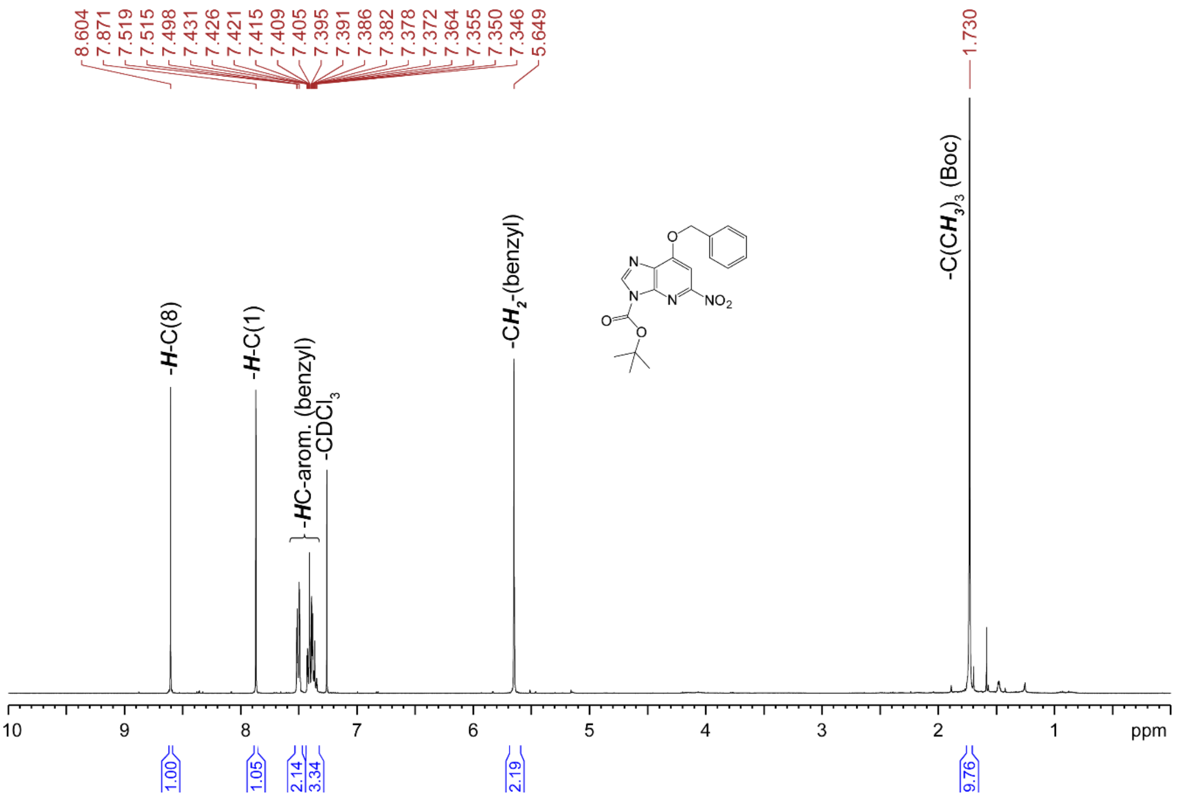

^{13}C NMR (100 MHz, $CDCl_3$, 25 °C) of compound **20**

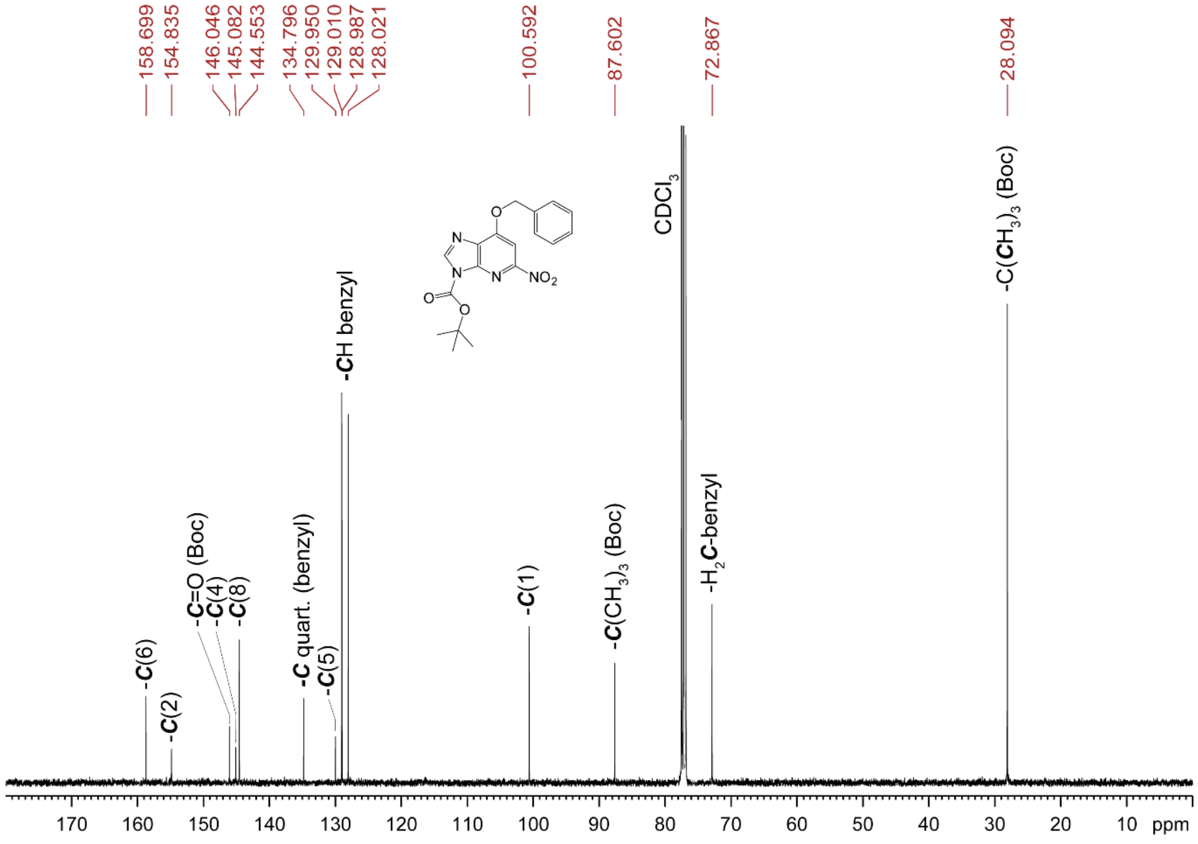

^{1}H NMR (400 MHz, DMSO-d_6, 25 °C) of compound **21**

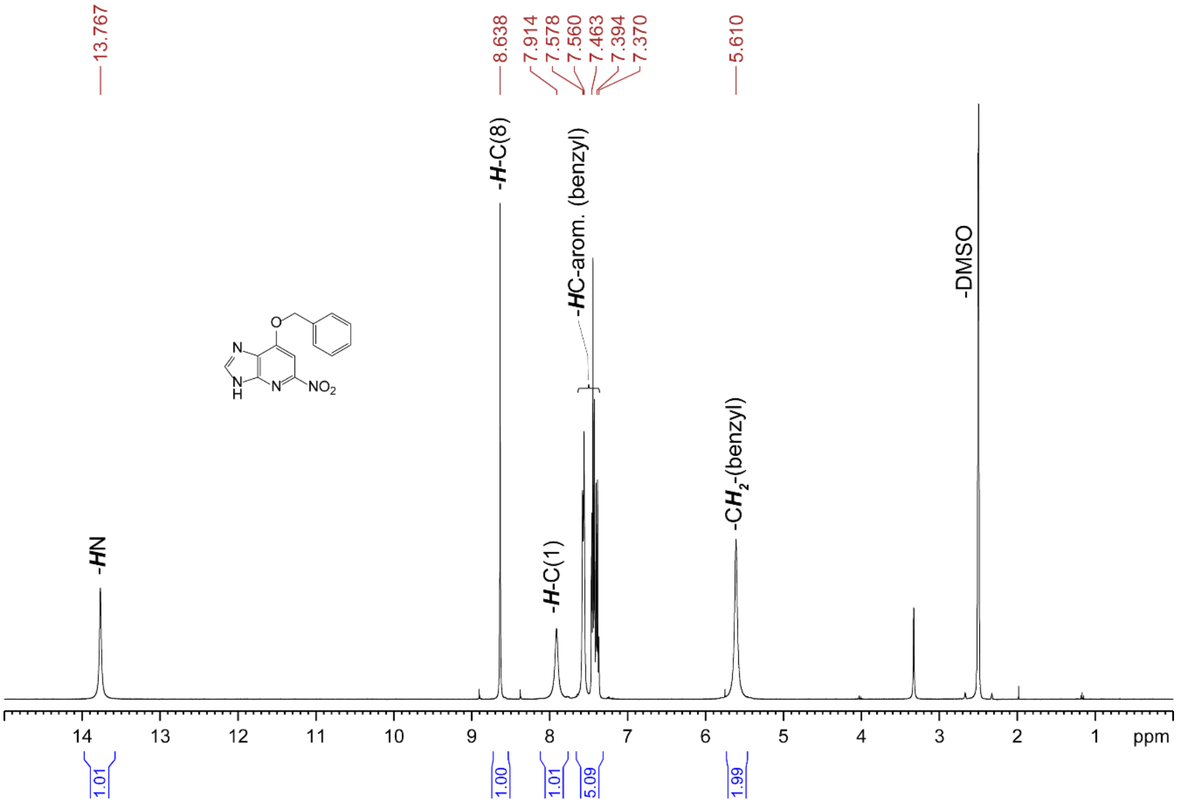

^{13}C NMR (100 MHz, DMSO-d_6, 25 °C) of compound **21**

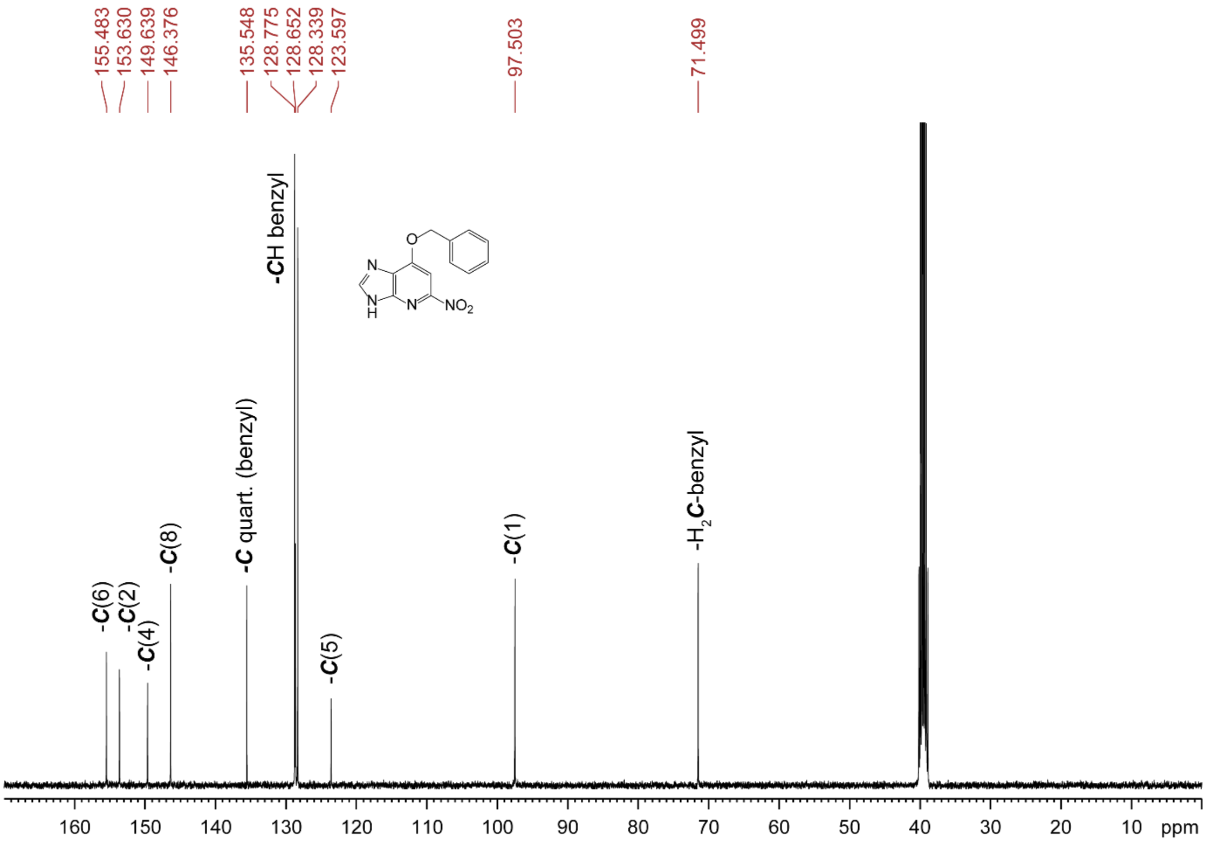

^{1}H NMR (400 MHz, DMSO-d_6, 25 °C) of compound **22**

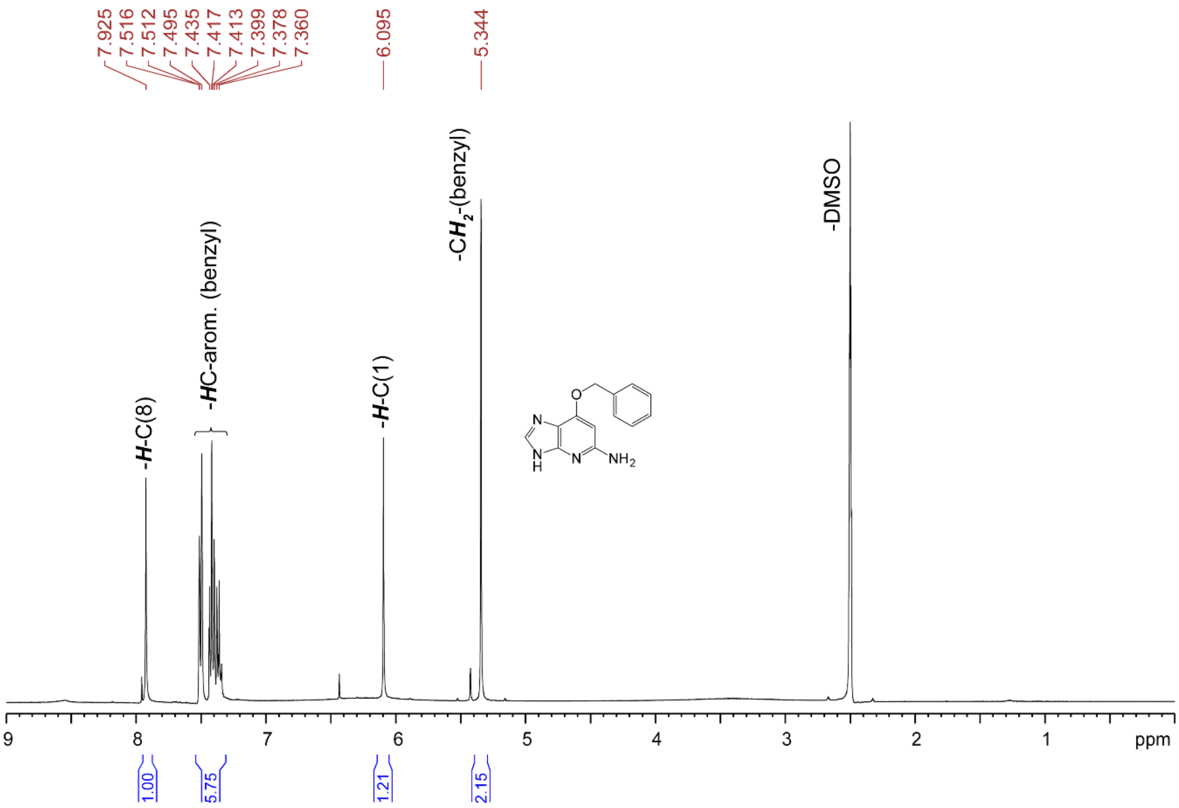

^{13}C NMR (100 MHz, DMSO-d_6, 25 °C) of compound **22**

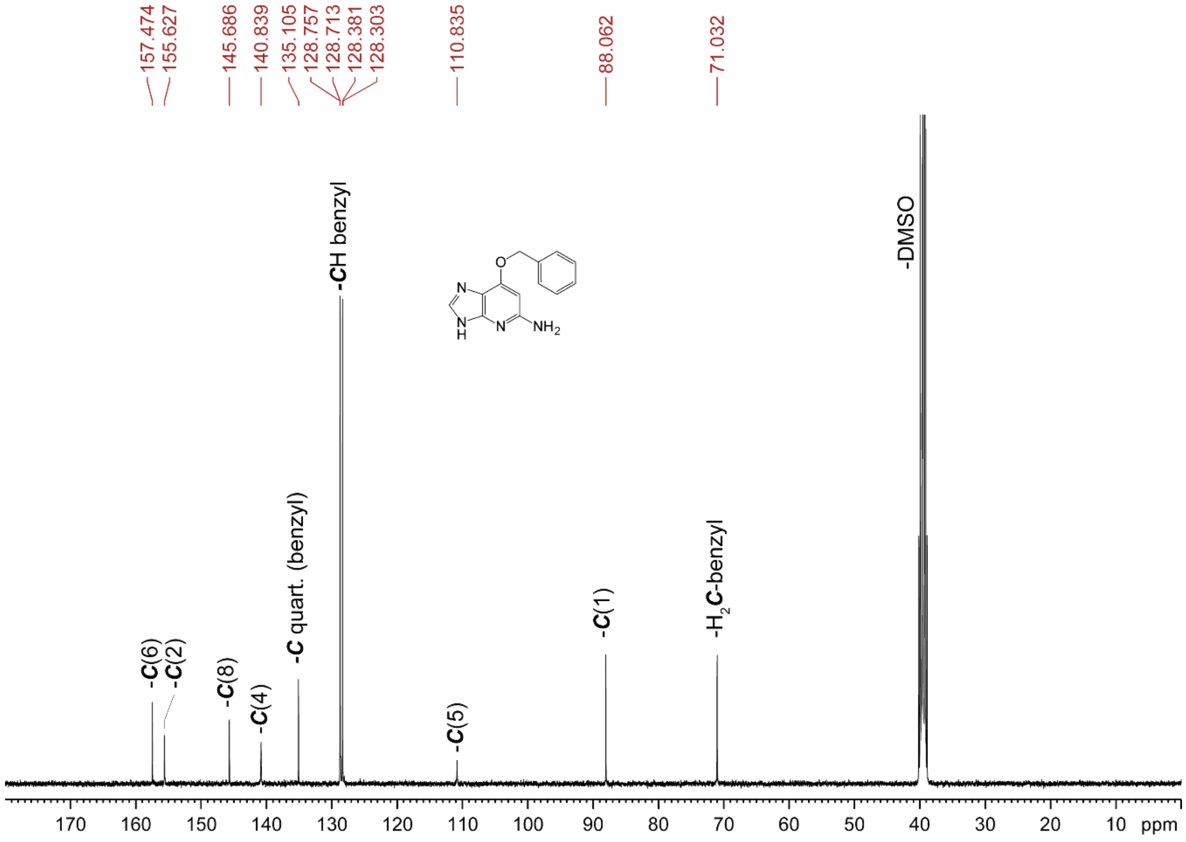

^{1}H NMR (400 MHz, DMSO-d_6, 25 °C) of compound **11**

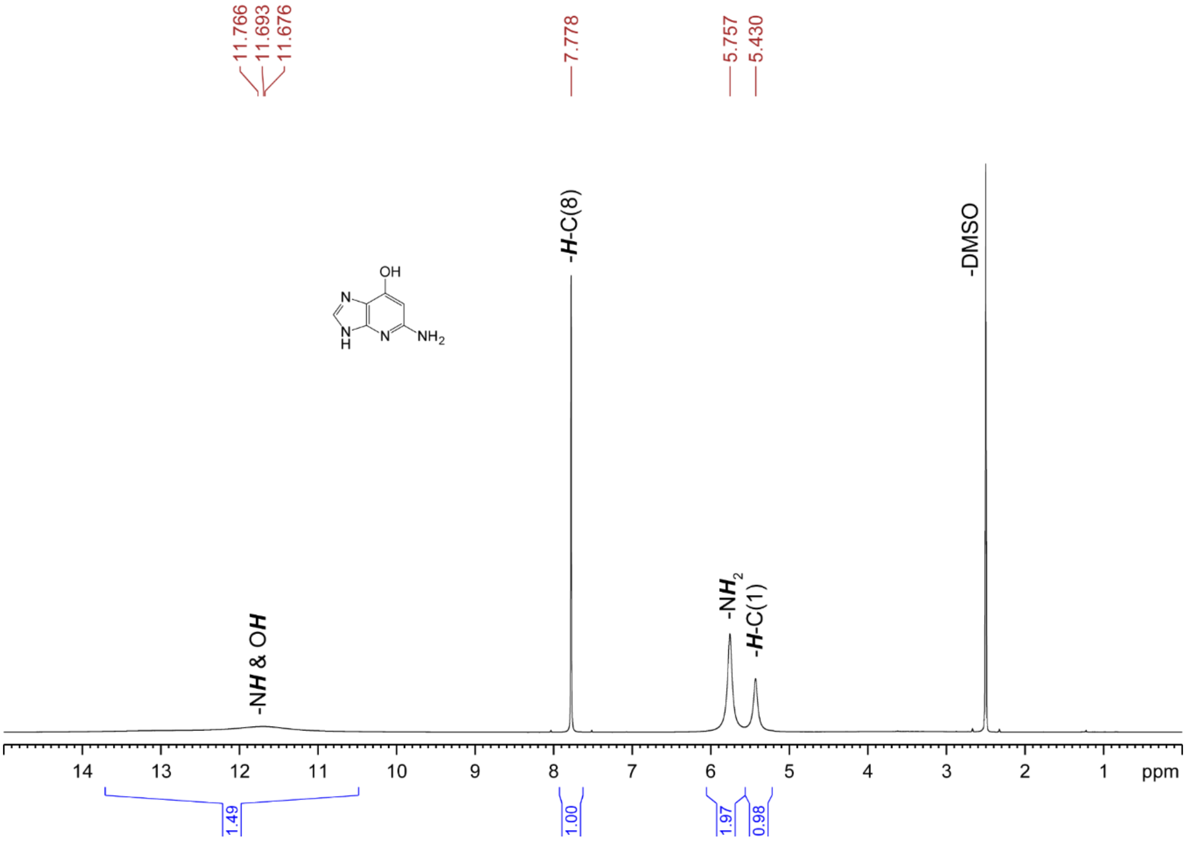

^{1}H NMR (100 MHz, DMSO-d_6 + 40 µl 5% HCl, 25 °C) of compound **11**

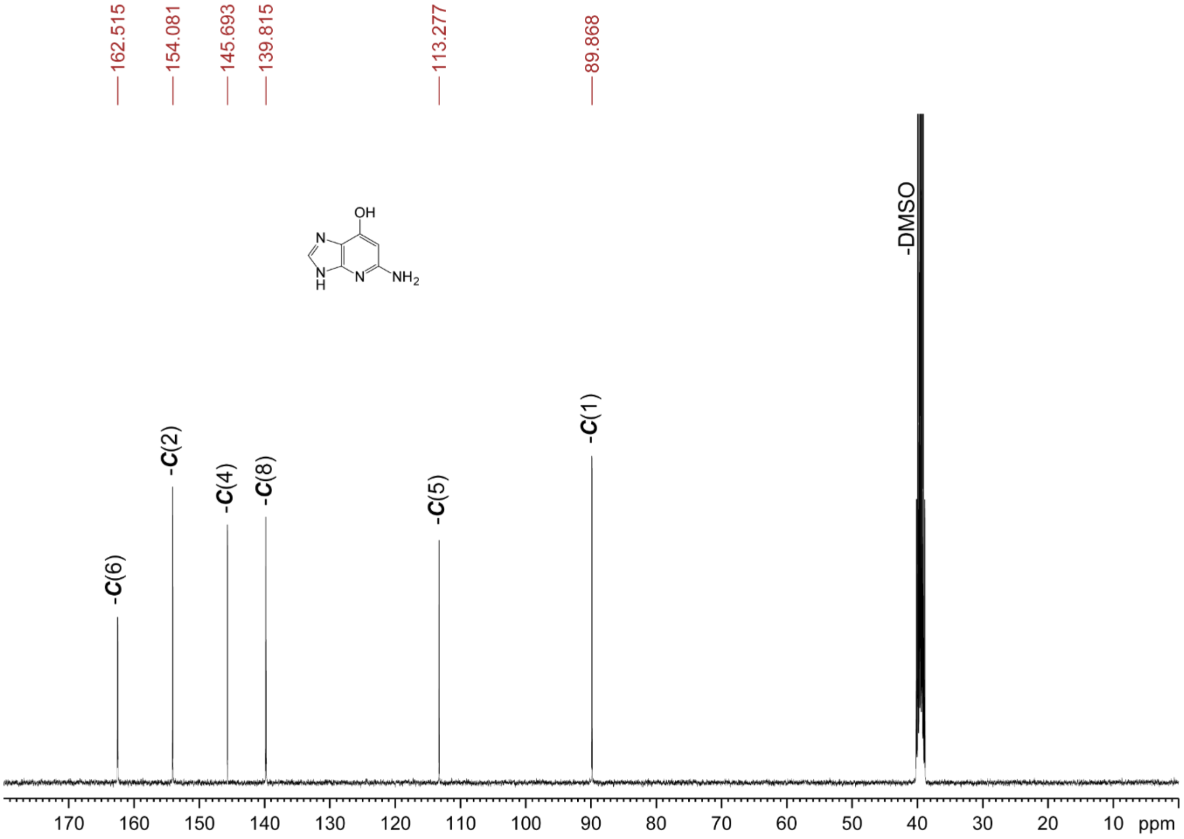

$^{1}H,^{13}C$ HSQC NMR (400 MHz, DMSO-d_6, 25 °C) of compound **11**

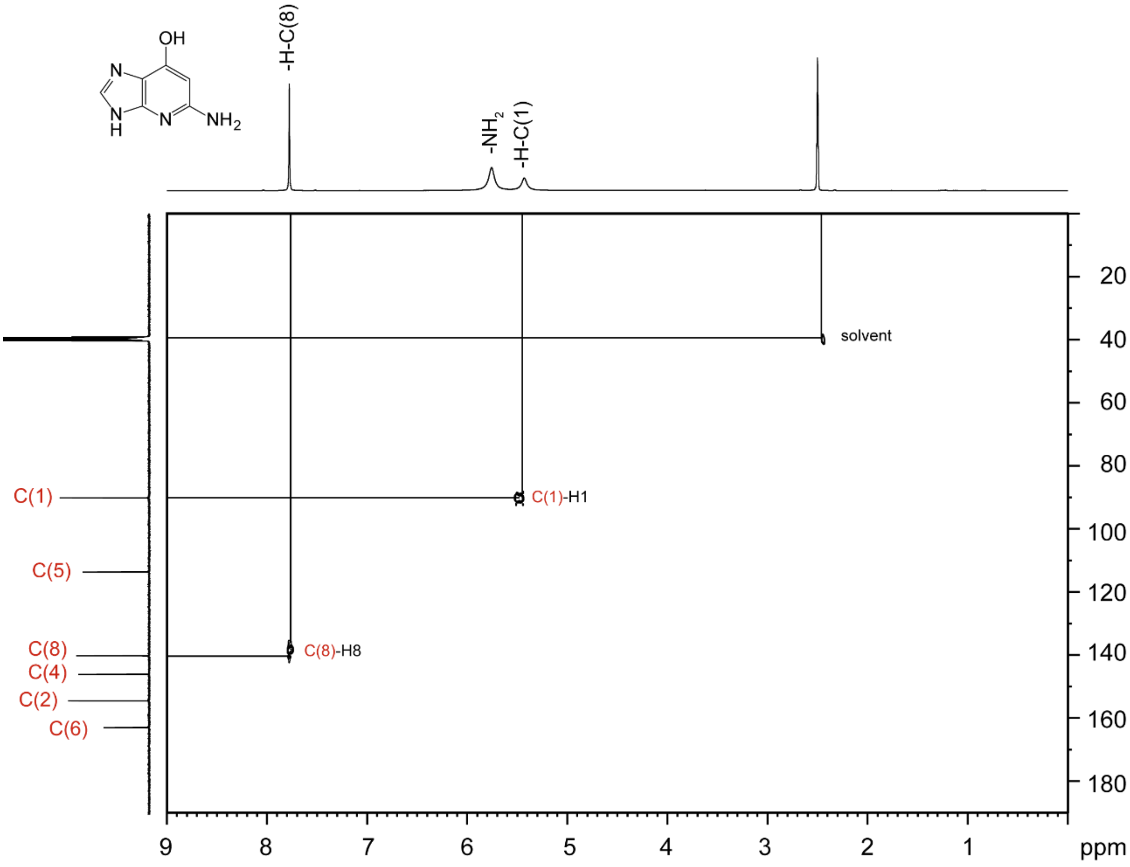

$^{1}H,^{13}C$ HMBC NMR (400 MHz, DMSO-d_6, 25 °C) of compound **11**

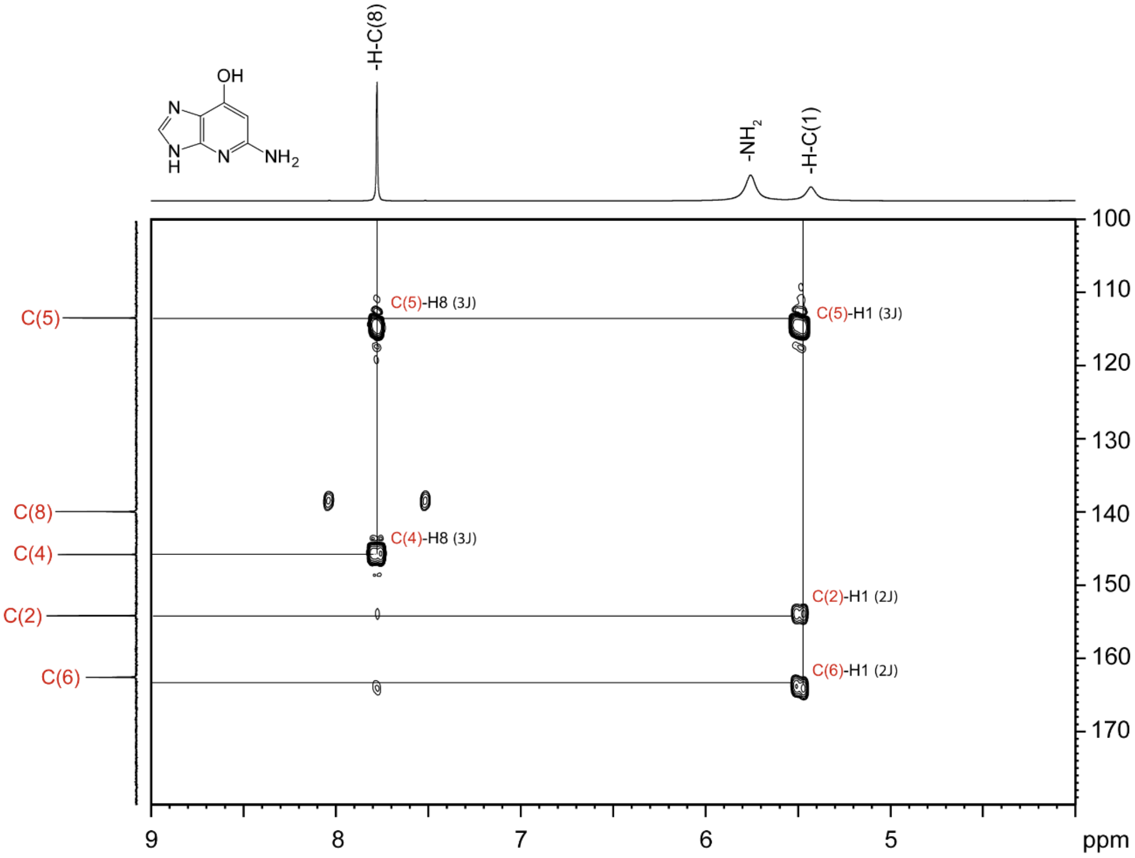

^{1}H NMR (400 MHz, DMSO-d_6, 25 °C) of compound **31**

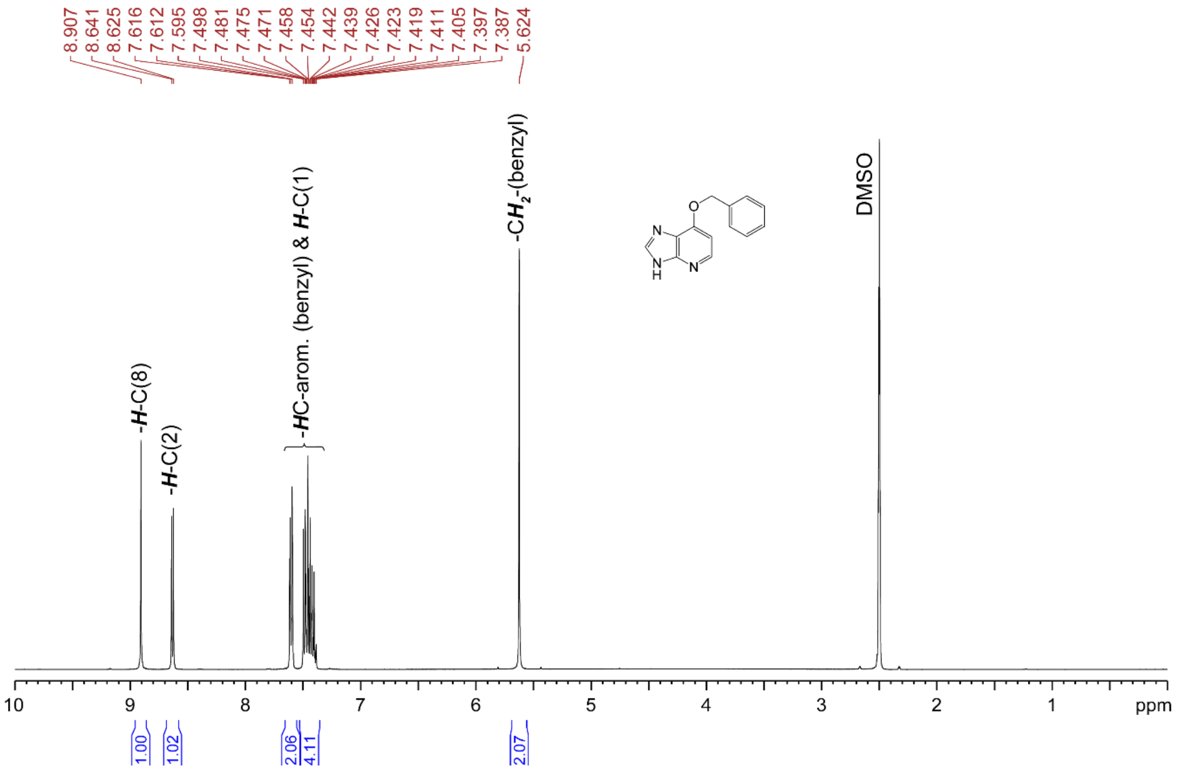

^{1}H NMR (400 MHz, DMSO-d_6, 25 °C) of compound **31**

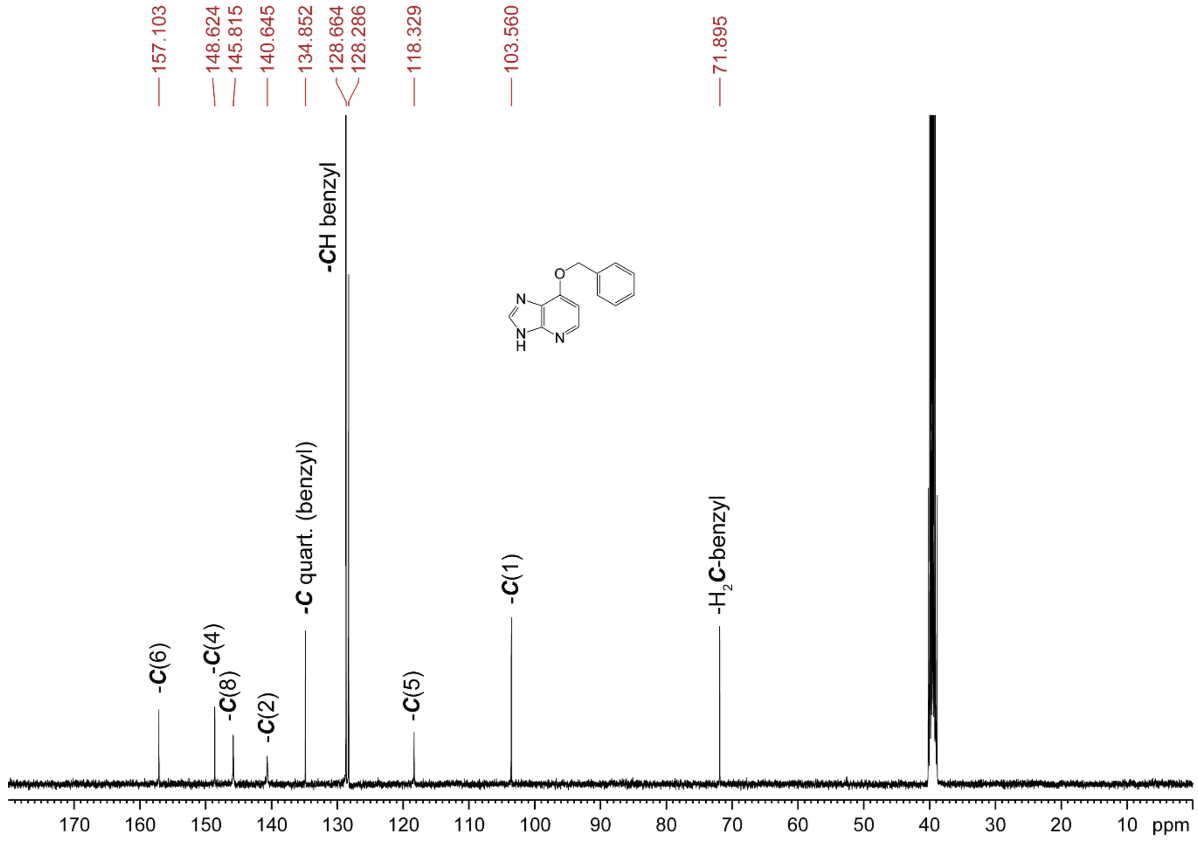

^{1}H NMR (700 MHz, DMSO-d_6 + 40 µL 5% HCl, 25 °C) of compound **30**

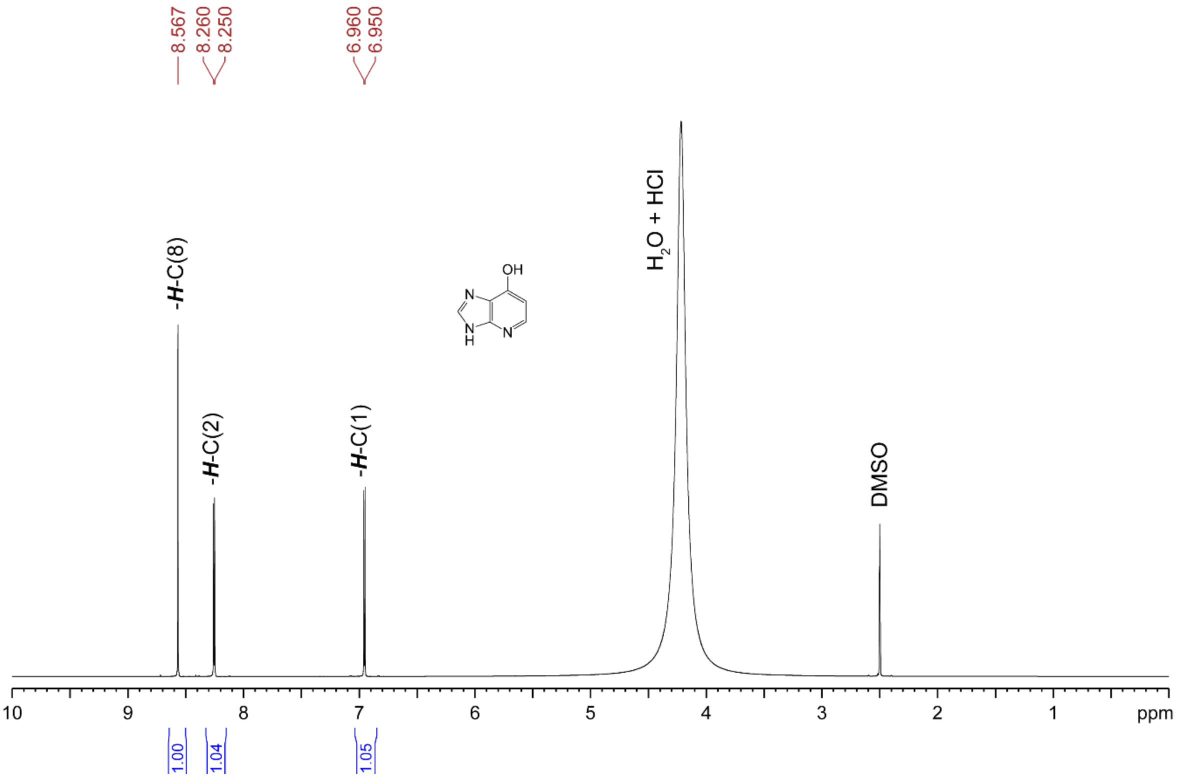

^{13}C NMR (175 MHz, DMSO-d_6 + 40 µL 5% HCl, 25 °C) of compound **30**

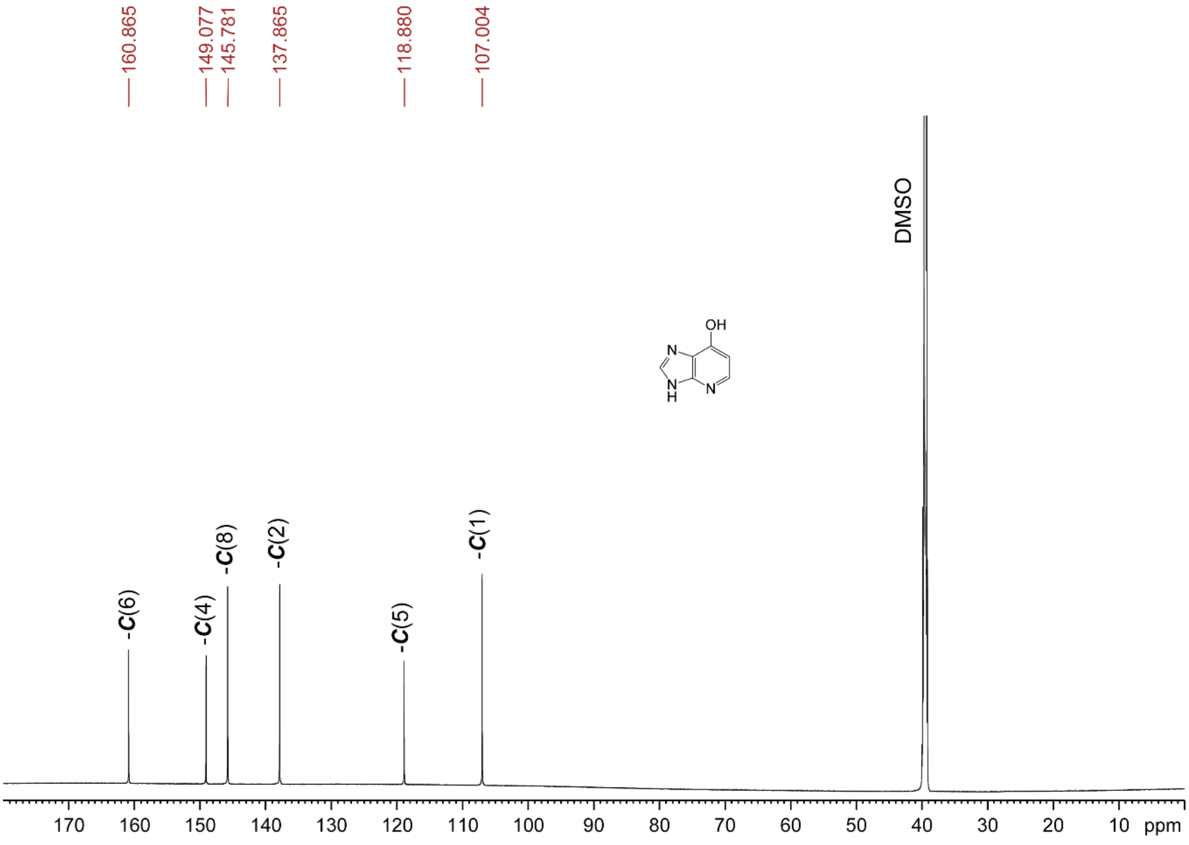

$^{1}H,^{13}C$ HSQC NMR (700 MHz, DMSO-d_6, 25 °C) of compound **30**

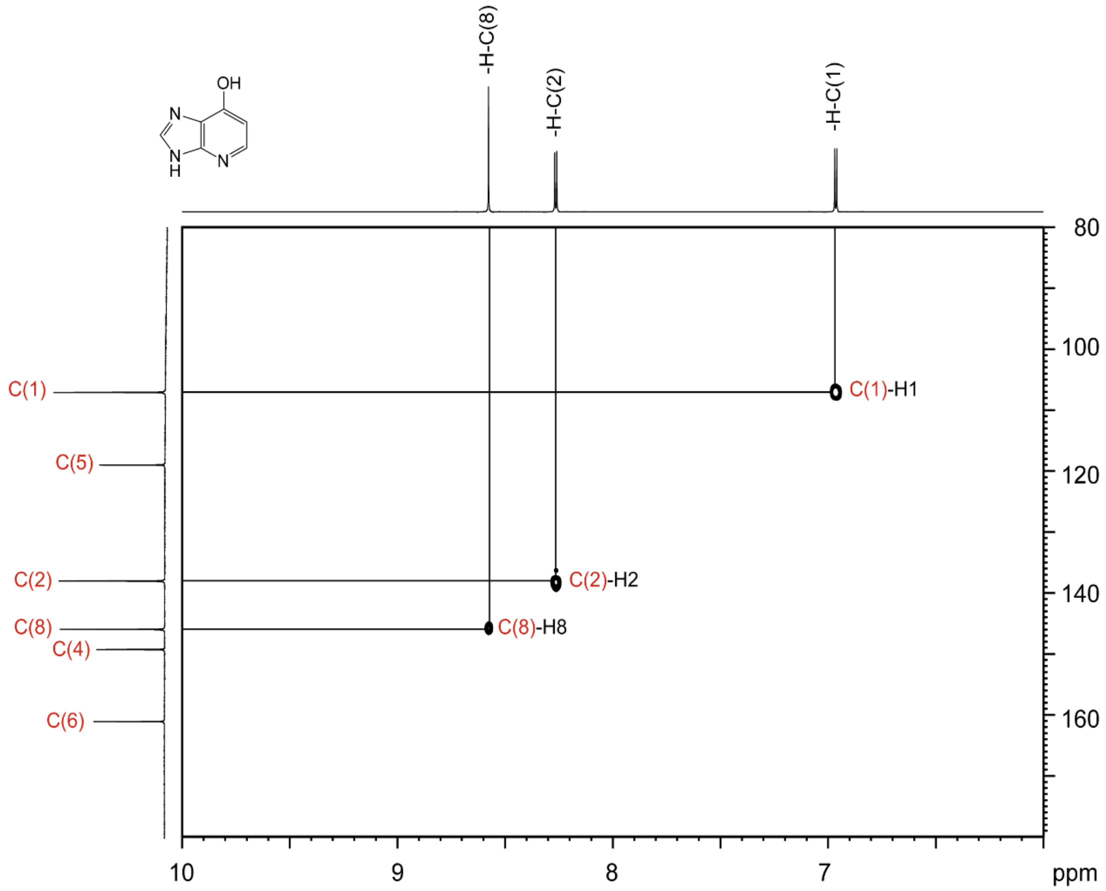

$^{1}H,^{13}C$ HSQC NMR (700 MHz, DMSO-d_6, 25 °C) of compound **30**

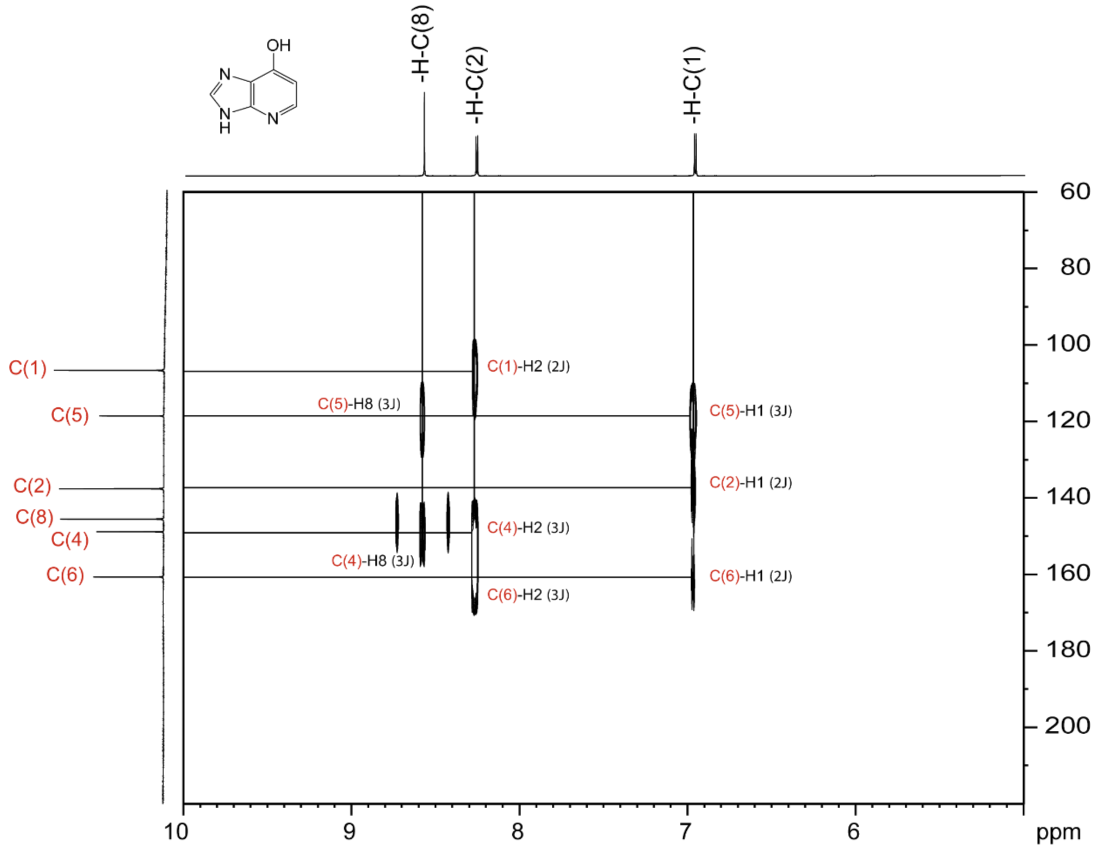

Supplement: File 1 — NMR spectra of compounds 17 to 22, 11, 31 and 30. [file Beilstein_J_Org_Chem-18-1617-s001.pdf]
